# Assessing the Psychosocial Impact of Expressive Writing on Adults With Spinal Cord Injury: Qualitative Study

**DOI:** 10.2196/71162

**Published:** 2025-06-30

**Authors:** Shelly M Xie, Molly McKenna, Kendall Veach, Sydney Williams, Mary Grace Jones, Elizabeth Vander Kamp, Salaam Green, Lauren Edwards, Kimberly Kirklin, Benjamin A Jones, Hon K Yuen

**Affiliations:** 1 Department of Occupational Therapy, School of Health Professions, University of Alabama at Birmingham, SHPB 354, 1720 2nd Avenue South, Birmingham, AL, 35294, United States, 1 2059346301; 2 Center for Arts in Medicine, University of Florida, Gainesville, FL, United States; 3 Arts in Medicine, University of Alabama at Birmingham, Birmingham, AL, United States; 4 Division of Neuroimmunology and Multiple Sclerosis, Department of Neurology, Heersink School of Medicine, University of Alabama at Birmingham, Birmingham, AL, United States

**Keywords:** narration, grief, internet-based intervention, health promotion, spinal cord injury, mobile phone

## Abstract

**Background:**

Spinal cord injury (SCI) results in significant physical, emotional, and social consequences, often leading to profound grief and unresolved emotional burdens. While expressive writing has shown potential in facilitating emotional processing and in aiding coping after trauma, loss, and health-related adversity, its impact on individuals who have suddenly lost physical abilities due to SCI remains underexplored.

**Objective:**

The study aimed to examine the experiences of adults with SCI who participated in a 10-week web-based coach-guided expressive writing program to understand its impact using a qualitative research design with a phenomenological approach.

**Methods:**

Participants were recruited through various outreach strategies based on a set of predefined criteria. A total of 50 individuals from 23 states expressed interest in the expressive writing program. Of those who met the eligibility criteria, 29 individuals completed the preprogram questionnaire. A total of 5 participants did not complete the postprogram assessments, including the exit interview. The program sessions were structured with reflective prompts designed to explore their emotions and life experiences related to their conditions. Qualitative data were collected through postprogram semistructured interviews and analyzed using thematic analysis to identify themes related to participants’ experiences and program impact. The analysis was conducted without any preset theoretical framework of reference.

**Results:**

A total of 24 adults with SCI, aged between 34 and 76 years (average age 51, SD 12 years), participated in the expressive writing program and the exit interview. Of these, 19 participants were White, and 17 were female. In total, 18 participants had sustained a traumatic SCI; of these, 10 had quadriplegia, 12 had paraplegia, and 2 had monoplegia. Qualitative analysis revealed three overarching themes: (1) supportive environment: the program provided participants a space that encouraged open reflection on past events and personal struggles, and the guidance of patient and empathetic coaches offered a sense of comfort, direction, and motivation. (2) Cathartic experience: the program helped them process complex emotions, reframe their perspectives, and cultivate a more positive outlook on life and their injury. Many participants, new to guided expressive writing, found the process therapeutic and transformative. (3) Acceptance of life: the cumulative impact of the sessions fostered self-compassion, forgiveness, empowerment, and self-advocacy. Participants reported reduced feelings of loneliness, a greater sense of community, and profound positive changes, expressing the desire to continue writing beyond the program.

**Conclusions:**

The sudden onset of lifelong disability due to SCI leads to profound physical, mental, and social challenges. The coach-guided expressive writing program enhanced the emotional processing and articulation, coping mechanisms, and overall well-being of the participants. These results highlight the potential of expressive writing programs as accessible and valuable rehabilitative interventions for individuals with SCI.

## Introduction

Spinal cord injury (SCI) not only results in physical immobility but also negatively affects social and occupational roles and personal life goals, leading to profound grief [1-4]. Grief, stemming from the life-long reminder of the loss associated with SCI, manifests as an intense emotional experience and significantly impedes improvements in functioning and overall health [4,5]. This unresolved emotional burden often resurfaces in response to complications from the injury or additional health conditions, architectural obstacles, or psychosocial stressors such as disability discrimination [4,6,7]. Consequently, individuals with SCI often perceive grief as a challenge they must navigate daily for the rest of their lives [3,8,9]. These significant psychosocial consequences highlight the pressing need for accessible, cost-efficient, and effective support programs that address the emotional needs of these individuals and facilitate their journey toward grief resolution [10,11].

Growing evidence suggests that creative arts interventions, including expressive writing, can alleviate emotional distress and facilitate the grieving process by helping individuals with chronic conditions adapt to their altered circumstances [12-16]. Expressive writing, a psychological intervention in which individuals explore and articulate their thoughts, emotions, and experiences in writing, is a means for internal processing and healing following traumatic or stressful life events [17,18]. It has been shown that expressive writing is particularly beneficial to individuals with physical illness, mental health conditions, or those experiencing trauma or loss [19-22]. Expressive writing enhances these individuals’ cognitive processing and self-reflection and strengthens their coping abilities [21,23,24]. A meta-analysis found that adults who engaged in expressive writing that focused on their feelings and emotions surrounding traumatic experiences exhibited a 23% improvement in various aspects of health, including physical and mental well-being and functioning, physiologic responses, and overall functionality, compared to those who wrote about neutral topics [25].

Although growing evidence supports the efficacy of expressive writing for facilitating emotional processing, promoting psychological well-being, and enhancing the coping mechanisms of individuals experiencing trauma and loss [17,21,23], limited research has explored its effects specifically in individuals who have suddenly lost physical abilities due to SCI [26]. A scholar who experienced an SCI published an account of his journey through injury rehabilitation and the stages of grief. He advocated for the therapeutic use of expressive writing to help individuals express emotions stemming from losses associated with limb paralysis “...to mourn their loss and make sense of their new lives.” [27].

Beyond the therapeutic and rehabilitative value, literature on disability studies has increasingly emphasized the importance of the arts in enhancing empowerment, agency, and identity formation, serving as a tool for challenging dominant ableist narratives and fostering self-expression [28-30]. Furthermore, community-based arts practices also demonstrate the transformative potential of creative engagement in fostering social connection, community building, and advocacy among individuals with disabilities [31].

This study explored how an expressive writing program not only provides a space and avenue for emotional reflection but also aligns with the broader movement of disability arts by offering participants an opportunity to reclaim their narratives and foster a sense of belonging. We conducted a 10-week coach-guided expressive writing program for adults with SCI [16]. We used qualitative methods to (1) gain a more nuanced and comprehensive understanding of participants’ perspectives on the expressive writing program and its impact on them and (2) refine program elements, including format, content, context, and appropriate evaluation instruments. Specifically, we analyzed interview responses from participants who completed the program to capture outcomes that we could not anticipate at the beginning of the program or measure by standardized quantitative outcome measures [32]. Therefore, the purpose of this study was to explore the experience of adults with SCI who completed a 10-week coach-guided videoconferencing expressive writing program.

## Methods

### Research Design

The 10-week expressive writing program (ie, the Writing for Healing workshop) was conducted via Zoom (Zoom Video Communications, Inc) videoconferencing with participants with SCI, individually or in small groups. After the program concluded, participants underwent one-on-one interviews. Interview data were analyzed and interpreted using thematic analysis, which constituted an essential part of the qualitative descriptive research design of this study.

### Ethical Considerations

This study was reviewed and approved by the institutional review board at the University of Alabama, Birmingham (300005546) and registered at ClinicalTrials.gov (NCT04721717) before participant enrollment. All procedures adhered to ethical guidelines for human participant research. All participants provided informed consent prior to their involvement in the study. To protect participants’ privacy and confidentiality, all study data were deidentified before analysis. Data were securely maintained and accessible only to authorized research personnel. Participants received a reloadable ClinCard as compensation for their time of up to US $100 for completing sessions and questionnaires.

### Participants

Participants were recruited based on the following eligibility criteria. Inclusion criteria were: (1) community-dwelling adults (aged 18 y or older) living with SCI, (2) able to communicate verbally or through writing, (3) able to access a computer or smartphone and home internet, and (4) able to understand the nature of study participation and provide informed consent. Exclusion criteria were: (1) congenital etiologies of nontraumatic SCI, (2) presence of cognitive or sensory deficits such as blindness or language barriers that could impede participation in the study and completion of its outcome measures, or (3) display of overt psychotic symptoms.

Participants were adults with SCI who were recruited through several avenues, including community agencies that serve people with physical disabilities; direct mailings to patients in a university rehabilitation center database; flyers posted on social media, such as Facebook groups for individuals with SCI and transverse myelitis; and word of mouth. Detailed recruitment procedures were previously reported [16]. A total of 50 individuals from 23 states expressed interest in the Writing for Healing workshop. Of those who met the eligibility criteria, 29 individuals completed the preprogram questionnaire. A total of 5 participants did not complete the postprogram assessments, including the exit interview, and 3 participants attended fewer than two expressive writing sessions.

### Procedures

Individuals who were interested in participating in the study contacted the research coordinator, who provided information about the study, screened individuals for eligibility, and obtained signed informed consent forms. After participants provided informed consent and completed the preprogram evaluation (ie, self-report questionnaires of outcome measures), they participated in the 10-week expressive writing program (1 h weekly sessions) guided by one of two professional teaching artists (ie, writing coaches) during scheduled Zoom videoconferencing sessions. To foster interest in writing, the videoconferencing coach-guided expressive writing program used various forms of writing, including affirmative, poetic, and transactional. The coaches introduced participants to new writing themes in each session. Themes included: (1) Emotional Disclosure with the prompt, “How do you feeling of living with an SCI?”; (2) Cognitive Appraisal with the prompts, “What does having an SCI mean to you?” and “What challenges have you overcome?”; (3) Benefit Finding with the prompts, “What has SCI taught you?” and “Are there ways in which you can use your experience to help others?”; and (4) Looking to the Future with the prompts, “What changes would you need to feel fewer negative emotions and more happiness and joy?” and “How have you dealt with this trauma?”

Once participants understood a prompt, they were given at least 20 minutes to write. After a short break, they were asked to read what they wrote with a spirit of wonder and curiosity as though they were reading a good friend’s writing. They could use the Zoom mute button to read their writing aloud to themselves if they chose. The intent was not to fix, edit, or alter their writing but simply to absorb what they wrote. Participants then engaged in reflective writing for 5‐10 minutes, responding to their initial writing. After completing reflective writing, participants were asked if they would like to share it with the group or the coach. Participants could share either or both the initial writing and reflective writing or not share any of their writing. Participants who did not share any writing usually summarized what they wrote. The writing coach facilitated an undirected supportive interaction if participants chose to share their reflective writing. Writing coaches documented their comments and reflections on participants’ writing immediately after each session. Postprogram exit interviews began in mid-January 2021 and ended in mid-January 2022.

Both writing coaches received training in guiding individuals in writing for health and incorporating trauma-informed arts practices. One writing coach (SG), a professional writer, storyteller, and literary healing artist, facilitated the writing-to-heal workshops. She is also a certified listener poet and has practiced in health care settings for more than 5 years, using writing and poetry as a modality for enhancing health and wellness. The other writing coach (EVK) is a certified compassionate listening facilitator-in-training who facilitates compassionate listening courses. She has facilitated writing classes for more than 2 decades, and for the last 6 years has offered writing, visual arts, and storytelling opportunities to patients at bedsides and in group settings at a local hospital. The coaches’ role in the program was to help participants express their emotions through writing on a specific theme (ie, writing prompts) and facilitate undirected supportive interactions in which group members shared their postwriting reflections.

Participants who were physically unable to use a device (keyboard, stylus, or pen) to write their stories used a speech-to-text software program to generate their writing. All participants were able to use an input device or a software program to write their stories without too much difficulty. At the end of each session, coaches encouraged participants to continue expressive writing on their own.

Upon program completion, participants were interviewed individually through Zoom about their experience and perceptions of the program (ie, the Writing for Healing workshop) by four occupational therapy graduate students or a research coordinator, all of whom were trained to conduct qualitative interviews. The interview questions focused on five topics: (1) reaction to the Writing for Healing workshop experience; (2) concerns, limitations, and difficulties related to the Writing for Healing workshop; (3) acceptability, satisfaction, and usefulness of the Writing for Healing workshop; (4) impacts on daily activities; and (5) suggestions for improvement (see Table 1 for the interview guide).

**Table 1.** Guiding questions for participants with spinal cord injury for interviews following the coach-guided videoconferencing expressive writing program.

Topic	Questions
Reaction to Writing for Healing workshop experience	How would you describe the Writing for Healing workshop experience? What was it like to work with the coach (and other participants) in the Writing for Healing workshop? What do you wish to get from this writing workshop?
Concerns, limitations, and difficulties of the Writing for Healing workshop	Describe the process of writing or storytelling in the Writing for Healing workshop (prompt level of frustration and fatigue). What do you like or dislike (ie, favorite or least favorite part) about the Writing for Healing workshop? Based on your experience, what were the difficulties or concerns you encountered in writing or storytelling during the Writing for Healing workshop?
Acceptability, satisfaction, and usefulness of the Writing for Healing workshop	How satisfied are you with the Writing for Healing workshop experience? How useful was the writing or storytelling in the Writing for Healing workshop? What was the most useful part of the Writing for Healing workshop for you? What aspects of the writing workshop have been particularly influential, positively or negatively, to you? What has been the best thing to come out of you attending the writing workshop? Are you going to carry on doing the emotional writing after the end of the writing workshop? Would you continue with this study if it was offered to you again? Would you recommend the Writing for Healing workshop to a friend?
Impacts on daily activities	Compared to before participating in the Writing for Healing workshop, how do you feel about yourself? Did the Writing for Healing workshop experience change you or affect your well-being? If so, please describe the change or effect (probe with questions about physical, social, emotional, intellectual, or personal impacts as needed).
Suggestions for improvement	What can we change to make the Writing for Healing workshop better? What suggestions or additional comments do you have for this writing workshop? Is there anything else you would like to share?

Interviewers adhered predominantly to the interview guide and suggested question sequence to facilitate discussion but could adjust the order of questions and incorporate additional probes to elicit responses based on each interview’s unique flow. Member checking was used actively during interviews through a process of reflecting and probing to verify that interviewers correctly understood what each participant said and meant [33]. Interviews were audio-recorded with participant consent and transcribed verbatim for thematic analysis. On average, the core of each interview lasted 15.6 (SD 7.4) minutes (ranging from 4.4 to 36.4 min). Participants were assigned numerical pseudonyms.

After completing the web-based expressive writing program, many participants expressed their desire to continue with the group writing program. Consequently, we organized a web-based evening gathering 3 months after the project’s completion. More than 75% (n=19) of participants who completed the writing program attended the 2-hour web-based gathering to share their experiences.

### Data Analysis

A phenomenological approach using thematic analysis was used to identify emerging themes from the interview transcripts related to participants’ experiences with the expressive writing program (ie, the Writing for Healing workshop) and the impact of these experiences on their emotions, moods, thoughts, and daily lives.

Two unique steps in the phenomenological approach, bracketing and intuiting, were implemented before data analysis [34]. Bracketing is a powerful strategy to minimize investigator biases [35] in which interviewers and coders bracket out their presuppositions and biases related to the study topic and content and approach the data with an open mind. To ensure data were approached neutrally, we selected interviewers and coders for this study who had no prior clinical knowledge of people with SCI or their experiences with expressive writing and had not conducted any literature searches on this topic before data collection (ie, interviews) and coding. By fully immersing themselves in participants’ perspectives, coders drew on their insights and understanding to grasp the essence of participants’ lived experience with the program (ie, intuiting). This attempt by coders to access the “meaning” behind participants’ words and descriptions of their program experience allowed a deeper interpretation of the data. The coders spent a full semester analyzing the data.

After verbatim responses from all participants were imported into a Word (Microsoft Corp) document, the qualitative data analysis was conducted using the following steps [36]. To gain an overall impression of participants’ responses and to formulate tentative ideas, the same 4 occupational therapy graduate students who interviewed participants read all transcripts independently and multiple times to fully immerse themselves in the data and participants’ perspectives. To generate initial codes (ie, open coding), they closely read each transcript, focusing on the content, context, and language used by participants. The coders ensured codes aligned with the original texts and preserved the participants’ meaning throughout the process. As analysis progressed, codes expressing related concepts were grouped into categories (ie, axial coding) that captured participants’ experiences. The coders then clustered categories into themes and subthemes by checking whether preliminary themes and subthemes were appropriate relative to the coded extracts and modifying them as needed (an iterative process) to ensure themes reflected the original data accurately. They integrated participants’ background information and writing coaches’ comments and reflections into the analysis as appropriate. Finally, coders defined and named the themes and subthemes. In addition, we presented our preliminary findings to participants who attended the web-based gathering held 3 months after the project’s completion. During the gathering, participants verbally shared their experiences with the expressive writing program. Information collected from the gathering served as member checking to verify findings and interpretations (ie, themes and subthemes). The main themes and subthemes that emerged from the data are presented in the Results section of this paper along with examples of participant quotations to provide additional context.

To reduce bias in the analysis and enhance credibility, the coders reviewed each other’s theme categorization, comparing and contrasting their findings. They met several times to check the plausibility of the data categorization and interpretation and discussed the meaning of themes and subthemes until they reached a consensus. To increase the rigor of the analytic process, the interpretations and derived meanings were constantly checked against the interview transcripts to verify that themes and subthemes reflected the participants’ actual words. When disagreements occurred, the team reviewed participants’ transcripts, and then discussed and resolved disagreements. To enhance the trustworthiness of transcript interpretations, an independent arbiter (HKY) with substantial experience in qualitative research and familiarity with the study served as an external auditor to verify the coding and emerged themes and subthemes.

## Results

### Overview

A total of 24 adults with SCI completed the 10-week expressive writing program and the exit interview; their characteristics are reported in Table 2. A total of 19 (79%) participants were White, and 17 (71%) participants were female. In total, 18 (75%) participants had sustained a traumatic SCI; of these, 10 (42%) had quadriplegia, 12 (50%) had paraplegia, and 2 (8%) had monoplegia. The mean age of the participants was 51 (SD 12) years (range: 34‐76 y). The mean year of postinjury was 17 (SD 13) years (range: 1.5‐52 y), and 6 (25%) participants had sustained their injury within 5 years of study enrollment. In total, 5 (21%) participants lived alone, and 10 (42%) participants received disability assistance.

Qualitative analysis of participant experiences with the program revealed three overarching themes with nine subthemes: supportive environment (overarching theme 1, with 3 subthemes); cathartic experience (overarching theme 2, with 2 subthemes); and acceptance of life (overarching theme 3, with 3 subthemes).

**Table 2.** Characteristics of participants with spinal cord injury who completed the coach-guided videoconferencing expressive writing program (n=24).

Name	Age (years)	Gender^a^	Race	Education^b^	Living situation^c^	Employment^d^	Diagnosis^e^	Injury level^f^	Postinjury duration (years)	Session type
P1	62	F	White	SC	WS	D	NS	P	4.4	Group
P2	60	F	White	PG	A	D	SCI	OLL	20.9	Individual
P3	44	F	White	SC	WS	HM	TM	OLL	3.1	Group
P4	73	M	White	PG	WS	D	SCI	Q	3.3	Individual
P5	34	F	White	PG	WS	FT	SCI	Q	11.8	Group
P6	50	M	Black	BA/BS	WS	D	SCI	P	6.5	Group
P7	59	M	White	V	WS	NW	SCI	P	15.8	Individual
P8	60	F	White	PG	A	D	SCI	Q	17.8	Group
P9	76	M	White	BA/BS	WS	D	SCI	P	33.2	Group
P10	43	F	White	PG	WS	FT	SCI	P	22.5	Individual
P11	49	F	White	PG	WS	PT	E	P	17.7	Individual
P12	53	M	White	BA/BS	WS	FT	SCI	Q	36	Individual
P13	45	F	White	BA/BS	WS	D	SCI	Q	29.3	Individual
P14	54	F	White	SC	A	PT	SCI	P	22.5	Group
P15	68	F	White	PG	WS	PT	TM	P	52	Group
P16	35	F	White	BA/BS	WS	D	SCI	Q	17.1	Group
P17	52	M	White	BA/BS	WS	FT	SCI	Q	34.6	Group
P18	39	F	Asian	BA/BS	WS	FT	SCI	Q	18.3	Group
P19	67	F	White	PG	A	R	TM	P	Missing	Group
P20	42	F	Black	SC	WS	D	SCI	P	16.3	Individual
P21	40	F	White	PG	A	PT	SCI	Q	6.4	Individual
P22	36	F	Mixed	BA/BS	WS	NW	SCI	P	1.4	Group
P23	49	F	White	BA/BS	WS	FT	TM	P	3.5	Group
P24	36	M	Black	<HS	WS	D	SCI	Q	3.3	Group

a Gender: F=female; M=male

b Education: PG=postgraduate; BA/BS =bachelor’s degree; SC=some college; V=vocation; <HS=less than high school.

c Living situation: A=alone; WS=with someone.

d Employment: FT=fulltime; PT=parttime; NW=not working; R=retired; D=disability; HM=homemaker.

e Diagnosis: E=ependymoma; NS=neurosarcoidosis; SCI=spinalcord injury; TM=transverse myelitis.

f Injury level: P=paraplegia; Q=quadriplegia; OLL=one lower limb.

### Overarching Theme 1: Supportive Environment

The Writing for Healing workshop created a supportive environment for participants by establishing an accepting environment and safe space (subtheme 1a), providing a supportive coach (subtheme 1b), and offering structured sessions (subtheme 1c).

#### Subtheme 1a: Establishing an Acceptance Environment and Safe Space

Participants reported that the sessions were a safe space for discussing uncomfortable topics and disclosing personal struggles associated with their SCI. Some sessions were held in a small group setting in which participants were unfamiliar with each other. This was not seen as a drawback, however, as indicated in this comment.

*I actually like being with someone else, hearing someone else talk allows you to hear someone else’s side. So it kind of like made it easier to be a little more open with your own issues or your own path when someone else is being open with theirs.*

[P16]

Given the safe environment, participants felt the sessions allowed them to look at past life events that they typically brushed aside. In addition, the writing coaches played a crucial role in creating an accepting environment where participants felt comfortable sharing their emotions and experiences. One participant stated:

*I sometimes came into the sessions, and I was really sad or down from the week I was having. I could write about that and share, and I always felt accepted when I didn’t feel like opening up. The coach did a really good job of making it okay for when I felt like that in a session. I felt like all my emotions were safe here and I could write about anything I wanted, and it was never dismissed*.

[P3]

#### Subtheme 1b: Providing a Supportive Coach

The coaches selected for the Writing to Healing workshop were highly praised by participants for their support and patience. For example, a participant noted that:

*Elizabeth, who was the leader of my group [ie, writing coach] did an exceptional job of explaining the rules, guidelines, and making us feel comfortable in an environment where you are willing to express things [emotions or injury specifics] that you may not be able to or willing to talk about in every type of environment.*

[P17]

Another participant noted:

*I just think she’s [Elizabeth, the writing coach] gifted in what I don’t know what how to name her skill set exactly but she just the way she interacted with us, just gave us liberty to say, or share anything, and put it out on the table. She didn’t hinder us or make us feel like that’s not important, or that’s not even what I was looking for. She just gave us a large space in which to pour things out that just bubbled up to the surface, depending on what writing prompt she would give us*.

[P15]

#### Subtheme 1c: Offering Structured Sessions

Participants also commented on the workshop structure and session duration. Each web-based session was led by a coach and lasted 1 hour. This format provided participants with clear guidance and motivation. The discussions led by the writing coaches allowed the participants to explore and discuss various emotions related to their SCI, such as feelings of depression, guilt, forgiveness, acceptance, pain, and success. Many participants said they approved of the duration of each session and the overall program. For example, one noted:

*I found that structure beneficial and it gave structure to what would have been an unstructured day, and I appreciated that*.

[P8]

The participants also noted that “there was always a set plan for the direction of the sessions,” (P10) and that “the structure was motivating” (P18). For example, a participant stated, “I appreciated the length [1 h] of the sessions because it didn’t feel like a burden” (P21). Another stated, “I enjoyed the length of time in each session, I thought it was beneficial to my week” (P4).

Regarding the appropriate timing for people with SCI to engage in a Writing for Healing workshop, one participant suggested:

*This [the workshop] should be a standard of care for anyone who suffered from a major injury or accident. Maybe not immediately because you are still processing [your injury] but definitely within a year of your accident*.

[P11]

Many participants also expressed their desire to continue writing as a means of healing after the workshop concluded.

### Overarching Theme 2: Cathartic Experience

The Writing for Healing workshop provided a cathartic outlet for participants by allowing them to experience therapeutic and insightful emotions (subtheme 2a) and gain a positive perspective and attitude on life (subtheme 2b).

#### Subtheme 2a: Experiencing Therapeutic and Insightful Emotions

The workshop was most participants’ first experience in a coach-led expressive writing program. Participants described the sessions as therapeutic. For example, one participant stated:

*I saw it as like a form of therapy, to be able to write your thoughts down, good, bad [and] mixed in or whatever, you just get it out and it’s sort of the same way, like when I’d be really stressed, and I’d write lists. And if I could put my list on paper quite often. I don’t come back to them, but it’s just the process of getting it out. That’s freeing.*

[P2]

Another described the writing process as “just therapeutic to be able to talk to someone and to write down your feelings and learn how to express thoughts in a different way” (P6). One of the writing coaches, EVK, further validated this by noting that, for this participant (P6), “[the sessions] pulled emotions out of him that he did not even know he had.”

Participants also discussed how the workshop helped them clarify some emotions about their injury or changed the way they viewed their emotions. For example, one participant stated:

*It [The Writing for Healing workshop] helped me to address feeling that I probably suppressed about having a spinal cord injury, along with other emotions and feelings that I was having. And by week 8 or 9, I felt like a burden was lifted off of me*.

[P20]

#### Subtheme 2b: Gained a Positive Perspective and Attitude on Life

The Writing for Healing workshop provided participants with opportunities to discuss past adversities related to their SCI in one-on-one web-based sessions with a writing coach or a small group setting. A participant stated that the sessions allowed her “… to reflect on the past in a way that was kind of guided in a naturally positive and accepting way. But that also gave me a chance to kind of reflect on my growth over the years” (P13). Participants appreciated the supportive environment the sessions created, which encouraged open discussion and sharing of strategies for coping with challenges. They found the workshop helped them cultivate a positive attitude and served as a tool for processing emotions, as illustrated in this comment.

*I sort of felt like, there was an art of the workshop. In the beginning, I feel like there were some tougher really emotional things. And by the end, they weren’t talking about those emotions as much, but it felt like we moved to a space of greater positivity. Like more forward-thinking and so that was something that I felt like that was the art of the workshop working through some emotions and then getting to a place of sort of thinking forward in life. I thought that was a really powerful way of running it and I really appreciated that. So, the prompts at the end, I felt like they were more imaginative towards the future as opposed to processing tough things in the past.*

[P21]

Participants discussed how the expressive writing sessions allowed them to reflect on life and provided unexpected benefits. Through the reflective writing prompts, participants talked about how the workshop helped them gain a new perspective on their injuries and the challenges in their lives. Some participants viewed the experience as an opportunity to gain clarity and reflect on the impact of their injuries. As one participant stated, the sessions forced them “…to look at the ins and outs of not just daily living, but how it’s [my transverse myelitis] impacted me how it has impacted my daily living, but also how it’s impacted me looking at things. How it’s impacted me and my relationships, how it’s impacted me in my relationship with myself and my viewing of myself” (P23).

### Overarching Theme 3: Acceptance of Life

#### Overview

The Writing for Healing sessions allowed participants to approach and accept different feelings within their lives, specifically, they felt less lonely and gained a greater sense of community (subtheme 3a), felt less stress (subtheme 3b), and were more forgiving of themselves and others (subtheme 3c), which enhanced their sense of self-advocacy and empowerment.

#### Subtheme 3a: Reduced Loneliness and a Greater Sense of Community

The cumulative effects of the Writing for Healing sessions were particularly beneficial for participants. They reported that, toward the end of the program, they felt less lonely and isolated as they connected with others who shared similar experiences. For example, one participant stated, “At one point I felt lonely, but now I don’t feel so lonely” (P24), and “I think I still have a lot to process, but I don’t feel as alone” (P22).

Participants also placed a high value on the sense of community fostered by this relatively brief 10-week workshop. For example, one stated:

*I think probably the most beneficial aspect of it to me was that it was with two other ladies who had the same diagnosis as I do so they have gone through some of the same struggles and hearing how it’s impacted them and how they’ve dealt with it and knowing you’re not alone and being able to support each other when sometimes those conversations and our writing got very emotional. And so having the other ladies there who have gone through similar stuff, I felt like that was a big important part of this healing process. In addition to the writing, but the fact that our writing got feedback in a positive supportive way*.

[P23]

Another noted:

*There were times when the other person (participant), would write something and then we would just start talking, and it wasn’t just Salaam [a writing coach] reflecting, but also, I would reflect and the other participant would reflect, and sometimes those conversations were just very meaningful for both of us.*

[P3]

This exchange with P3 was followed by the interviewer, the research coordinator, asking, “That’s great. And so, is it correct if, tell me if I’m wrong, but is it correct in saying that you felt like there was a sense of community?” P3 responded, “Absolutely.” The sense of community and shared experience also contributed to participants’ sense of empowerment. The participant continued, “I view life as more capable now. I am more open to sharing my truth [transverse myelitis] and feel more free to express myself” [P3].

#### Subtheme 3b: Feeling Less Stressed

Throughout the workshop, participants viewed the Writing for Healing sessions as opportunities to talk freely and enjoy a sense of community. Many participants, depending on how recently their SCI had occurred, were still dealing with daily physical and emotional stress. A participant noted, “…it [the sessions] allowed me to escape from life’s adversity” (P11). Participants also noted that they experienced less stress and an increased ability to advocate for themselves after completing the program. One participant said:

*People would ask me things which would increase my stress about my injury, but now when I am asked about myself, I am able to answer them, and I feel more comfortable with being able to be vocal about certain things that I wouldn’t have before.*

[P16]

#### Subtheme 3c: Feeling Forgiving of Themselves and Others

When participating in the Writing for Healing workshop, some participants felt that they could forgive people who had hurt them in the past or did not understand their diagnosis and the complications that arose from it. A participant noted:

*I think I’ve identified some, some things and it surprised me, especially with the forgiveness prompt. I was like, oh, wow, you know, I have a lot of people that I am really angry about. It was it was kind of a revelation like, well, man, I’m holding a lot of negativities. And I think, you know, I feel lighter, I guess after writing for that prompt and doing a second one around the same kind of topics*.

[P18]

Participants also expressed that they were better able to open their minds to new thoughts and forgiveness of others and themselves at the end versus the start of the workshop. For example, one participant noted:

*Well, sometimes my mind kind of just looks at the past and picks very specific things to focus on to tell a narrative and it’s not that great sometimes. You know, especially when it revolves around trauma, and I think that this helped me look at it with kind of a bird’s eye view or allowed me to give myself some grace and forgiveness and not be so hard on myself*.

[P13]

## Discussion

### Principal Findings

The sudden onset of lifelong disability caused by SCI often results in profound physical, mental, and social challenges. This study demonstrates that the coach-guided videoconferencing expressive writing program is a promising approach that can help adults with SCI gain a positive perspective and attitude toward life and experience therapeutic and insightful emotions, facilitating their journey toward grief resolution. Overall, participants experienced profound, positive changes resulting from participating in the expressive writing program, highlighting its transformative impact. A high percentage of participants completed the 10-week writing program and attended the postproject web-based gathering, which indicates they felt that the coach-guided expressive writing program added value to their lives.

The coach-guided expressive writing program provided a supportive environment in which participants with similar experiences felt less lonely and isolated. Participants in small group settings encouraged and supported one another, highlighting the importance of acceptance and establishing a safe space to discuss personal challenges related to SCI and share coping strategies. The two writing coaches strictly adhered to the expressive writing program protocol, which included weekly themes or activities and writing prompts regardless of whether sessions with participants were conducted individually or in small groups. However, participants in small groups had the opportunity to share postwriting reflections with others, which reduced feelings of loneliness and increased their sense of community (subtheme 3a). This unintended positive consequence could not have been achieved had we conducted all sessions individually.

The emphasis on creating a supportive environment and the role of the coaches in guiding participants through the writing process resonates with the importance of interpersonal dynamics in therapeutic interventions [24]. This study’s supportive environment emerged as a pivotal aspect of the intervention, as engaging with peers facilitated a shift toward a more positive outlook on life. The participants reported that, by confronting their past, making sense of their losses, and embracing their injury-related limitations, they experienced decreased distress and an enhanced sense of meaning and purpose.

Findings from this study also echo the broader discussion in disability studies on how the arts play an integral role in individuals’ cultural and social lives, shifting from a solely clinical perspective. The participants’ descriptions of increased confidence, empowerment, and sense of agency align with findings that artistic expression serves as a platform for self-advocacy and representation [37]. Additionally, the expressive writing program fostered a shared space where participants felt validated and connected to others with similar experiences, reinforcing the significance of community-oriented artistic engagements [31,38].

Central to the program’s success was the creation of a safe space by the coaches, who demonstrated patience and attentive listening. Their guidance enabled participants to overcome their inhibitions gradually and engage in the writing process wholeheartedly. The participant’s acceptance of the coaches was crucial to the program’s effectiveness. Participants in previous coach-led expressive writing programs have also emphasized the value and impact of writing coaches in helping them explore their emotional states and improve their ability to express themselves and their emotions regarding important personal health issues [39-41]. Moreover, many participants expressed a desire to continue expressive writing beyond the program’s duration, underscoring its value as a long-term resource for healing and self-discovery.

Qualitative analysis of participants’ exit interview responses revealed that the expressive writing sessions were a cathartic experience, enabling them to articulate and release their thoughts and emotions and engage in introspection and self-expression. Furthermore, participants noted a shift toward a positive perspective on life, indicating the program’s potential to foster resilience and optimism in the face of adversity. The findings of this study align with existing literature on the benefits of expressive writing interventions for individuals facing adversity (ie, trauma and loss), including physical illness and mental health conditions [17,19-22,40]. The expressive writing program may have encouraged participants to rethink their behaviors, leading to changes in cognitive processing and coping mechanisms, as demonstrated in other studies on expressive writing [24,42].

The theme of acceptance of life further elucidated the program’s impact on participants’ emotional well-being. By fostering a sense of community and connection, the program helped alleviate the feelings of loneliness and isolation commonly experienced by individuals with SCI. Additionally, participants reported reduced stress levels and an increased capacity for forgiveness toward themselves and others. These findings highlight the transformative potential of expressive writing in promoting emotional resilience and self-compassion in individuals with SCI. Notably, participants reported enhanced self-advocacy and empowerment as a result of completing the program. Many expressed newfound confidence in discussing their diagnoses and sharing their stories, demonstrating the program’s broader impact on their sense of agency and self-expression.

As this was a pilot study, we did not exclude people with SCI based on the duration of their injury. Although psychological support may be more beneficial to those with a recent injury compared to those with an injury of longer duration [43,44], all our participants had sustained their injuries more than a year before entering the study. Most individuals with SCI in the subacute stage would likely not have been mentally prepared to participate in this program, which was not part of clinical rehabilitation and was conducted in a home setting. This pilot program demonstrated a high degree of success for individuals with SCI and can offer them a complementary or alternative avenue of psychological intervention. Therefore, our next step is to refine the program for scalability to support individuals with other conditions that involve a sudden loss of physical abilities, such as stroke, brain injury, and amputation.

Although participants reported an overall positive experience with the program, several initially expressed concerns about its 10-week duration. At the outset, some felt that 10 weeks was too long, however, by the program’s end, many felt it was not long enough. A proposed strategy to encourage enrollment of people with SCI is to advertise the program as a 5-week program while offering the option of a 10-week extension. In addition, scheduling group meetings with members who resided in different time zones was challenging. As a result, some participants met with the coach individually. Finally, some participants had unstable internet connections that led them to reschedule sessions. To accommodate group members who missed sessions due to scheduling conflicts, the coach provided individual make-up sessions within the same week to ensure they were prepared to rejoin the following group session.

### Limitations

This study’s sample size was limited and predominantly included highly educated, non-Hispanic White women, which may affect the transferability of the findings. Further research is needed to explore the implementation of the expressive writing program across diverse demographics and settings and advance the understanding of effective interventions for individuals with SCI. Since the interviews were conducted immediately after the program ended, this study’s findings reflect only the immediate perceived impact of the program. Future studies may consider conducting interviews twice, immediately after program completion and several months later, to explore both immediate and sustained benefits of the expressive writing program perceived by participants.

As we did not have permission to video-record writing sessions or access participants’ writing, we could not use transcripts and field notes to examine group dynamics. Instead, we relied on the writing coach’s reflections on the nature of interactions (between coaches and participants and among participants) to understand the group communication process and its contributions to participants’ insights and feedback about their experiences. The purpose of this investigation was to understand participants’ perspectives on the expressive writing program and its impact, rather than their emotional reaction to their SCI. Therefore, analyzing participants’ actual writing (initial and reflective) completed during the program would not add significant value to the research findings. Additionally, if participants had expected that their actual writing, which included personal health information, would be shared with and read by unknown individuals, they would have been less likely to express their emotions freely, defeating the program’s purpose.

### Conclusions

In conclusion, the coach-guided expressive writing program has emerged as a valuable tool and an achievable avenue for individuals with SCI to process and alleviate grief and stress across various dimensions. The results from this study suggest that the program promoted psychosocial adjustment and well-being in individuals living with SCI, which could ultimately strengthen coping abilities and resilience in the face of adversity. This study demonstrates the feasibility and potential efficacy of a coach-guided expressive writing program specifically tailored for individuals with SCI, addressing a critical gap in the literature on rehabilitative interventions for this population. The study provides an important lens for understanding the role of nonclinical programs and services in the lives of people with SCI. While the study’s limitations should be acknowledged, the findings hold promise for further exploration to extend these results to diverse populations with limb paralysis.

## Abbreviations

SCI: spinal cord injury
